# Social ties drive post-fission group choice in blue monkeys

**DOI:** 10.1098/rspb.2025.0376

**Published:** 2025-09-24

**Authors:** Rory Wakeford, Marina Cords

**Affiliations:** ^1^ Department of Ecology, Evolution, and Environmental Biology, Columbia University, New York, NY 10027, USA

**Keywords:** animal sociality, decision-making, permanent group fission

## Abstract

Permanent group fissions present rare opportunities for socially philopatric individuals to select their groupmates. We hypothesized that animals prioritize maintaining beneficial social ties during fission. We assessed post-fission group choice in philopatric adult female blue monkeys by considering the strength and consistency of their social ties, dominance relations and relatedness with female peers, as well as their risk of infanticide and ties to the original group’s resident male. Using a temporal network model, we assessed which females remained together (i.e. maintained ties) after fission. Females preferentially maintained ties to individuals with whom they had consistently strong affiliative ties before fission. More closely related individuals were likely to maintain ties only if they had similar risks of infanticide. Conditional logit models showed that females vulnerable to infanticide were more likely to join the post-fission group with the original group’s resident male than the group without him. Overall, female blue monkeys appear to consider multiple types of relationships when structuring their post-fission groups, prioritizing consistent affiliative ties with other females and ties with a familiar male, and even separating from kin when infant offspring are vulnerable. The relationships prioritized during fission likely confer benefits, and females’ choices illuminate how sociality influences decision-making.

## 1. Introduction

Social philopatry, the tendency for certain animals to remain in their natal group for life, confers both costs and benefits to group-living animals. Philopatric animals benefit from long-term associations and cooperation with kin and avoid the costs of dispersal related to seeking out unfamiliar territory or integrating into a new social group [1]. However, remaining in one’s natal group can impose costs if group size and resource competition increase. Philopatric animals have limited flexibility in choosing the size and composition of their groups, which means they could end up living in a social arrangement that does not optimize their survival and reproductive success. Social philopatry has been documented in many mammal and bird species and is typically sex biased (one sex disperses while the other remains in their natal location), theoretically according to which sex receives the greatest fitness advantages from staying in the natal group [2].

In social species with sex-biased philopatry, group fissions, the process by which a group of animals permanently splits into two, may be an important mechanism for regulating group size and composition [1]. Prior research has shown that post-fission group choice is consistent with egalitarian decision-making, with each individual influencing the post-fission arrangement [3]. Individuals should select a new group after fission that benefits their survival and reproductive success, allowing them to maintain advantageous social ties, which may vary depending on the species’ social organization. In matrilocal species, in which females are the philopatric sex, maternal kin tend to stay together after fissions [4–7]. The opposite trend is evident in patrilocal species, where males tend to stay after fission with paternal kin [8] or male groupmates that were previously preferred affiliates [9]. However, not all species prioritize maximizing genetic relatedness in post-fission groups [10]. In patrilocal muriquis, males choose post-fission groups to optimize the male:female sex ratios of each group [11]. In matrilocal moor macaques, reproductive status influences post-fission group choice, as non-cycling adult females preferentially associate with each other [12].

By examining how animals choose their new groups after permanent fission, we also gain insight into how they make complex social decisions with long-term fitness consequences. Most research to date, however, has focused on the factors influencing temporary fission–fusion dynamics. These factors include genetic relatedness [13–16], dominance rank [17], sex [18–20], mate selection [21], shared space use [18,19,22], reproductive status [14,23,24] and social affiliation [15,25]. However, the factors that influence temporary associations are likely to differ from those that influence permanent group choice. Fission–fusion dynamics reflect more transient and flexible decision-making processes in response to changing environmental conditions [26] and they do not permanently sever ties between groupmates. Group choice after permanent fission is a more consequential decision for an animal’s fitness.

Previous studies evaluating which social factors influence permanent post-fission group membership have either focused predominantly on a single factor (often relatedness) [5,6,8–10] or examined only a subset of potentially relevant social factors [4,7,11,12]. However, individuals living in groups navigate multiple types of relationships that can simultaneously influence their fitness. Additionally, individuals may vary in terms of which kind of relationship most enhances their fitness based on their individual attributes; for example, individuals without close maternal kin may prioritize ties to non-kin or paternal kin [4] or may venture off alone [10]. Therefore, it is important to analyse an array of potentially relevant social factors at once and to account for the possibility that animals will vary in their decision-making strategies depending on their individual attributes.

We assessed post-fission group choice in blue monkey adult females (*Cercopithecus mitis*), aiming to illuminate which of their ties they prioritize, which should be those that are most advantageous in a group-living context. Blue monkey females are philopatric, living in stable one-male groups, usually for an entire lifetime. Affiliative social ties are strongest among the closest maternal kin [27,28]. Studies of other cercopithecine primates have demonstrated that sociality is associated with improved survival and reproductive success [29,30]. These benefits have also been observed in blue monkeys in certain circumstances, such as when an adult female has strong and consistent affiliative ties with peers [31]. Adult blue monkey females have hierarchical agonistic relationships, with higher ranking females experiencing preferential access to fruits during specific reproductive phases [32]. However, dominance rank does not predict adult female survival [31], fertility [33] or nutrient balancing [34]. Males are the dispersing sex and most groups have only one resident adult male at a time. However, influxes of adult males occur regularly, especially during the breeding season and in larger groups [35], sometimes leading to male takeovers. Infants are at risk of infanticide from newly arrived males [36]. Females can potentially improve their fitness by affiliating with males that provide protection from infanticide, harassment and predation [ 37, 38].

We hypothesized that blue monkey post-fission group choice is non-random, favouring the persistence of advantageous pre-fission ties with social affiliates, kin and the resident male from the original group. We predicted specifically that females would stay with their closest and most consistent social affiliates. Strong, consistent ties are associated with increased longevity in this species; by contrast, females who change their most strongly bonded affiliates from year to year have a higher risk of death, which even exceeds that of females with weak ties [31]. Therefore, it could be detrimental to a female’s survival to join a post-fission group without her closest social affiliates. We predicted accordingly that females in pre-fission dyads with high social tie strength across the 2 years before fission would remain together after the group split, whereas weakly tied partners or those with inconsistent tie strength would be less likely to remain together, with inconsistent ties having the lowest likelihood of persisting. We also predicted that females would stay with their kin. Living with kin facilitates enduring cooperation and tolerance among relatives, enhancing an individual’s inclusive fitness [1,28,31,39].

Additionally, female dominance rank has been linked to several metrics related to survival and reproduction in social mammals, including many cercopithecine primates [40]. Therefore, in species with hierarchical social structures, females can potentially improve their fitness by joining a post-fission group in which they are higher ranked [41]. In this case, one would expect females with disparate ranks to be less likely to join the same group, as lower ranking females avoid the group with their superiors. Because rank has a minimal effect on blue monkey fitness, however, we predicted that rank would not affect which females stayed together after fission, and particularly that ties between females closer in rank would not be more likely to persist after fission than ties between more disparately ranked females.

Finally, given the risk of infanticide from novel males [36], a female can also influence her reproductive success by staying with the original group’s resident male if she has a dependent infant. Therefore, we predicted that females who were at risk of infanticide—pregnant or nursing a young infant—at the time of fission would tend to select the post-fission group that contained the original group’s resident male as an anti-infanticide strategy. We also examined whether the strength of a female’s affiliative tie to the original group’s resident male increased her likelihood of selecting the post-fission group he was in.

## 2. Methods

### (a) Study population

This study included data from five instances of group fission observed between 2008 and 2019 in the blue monkey (*C. mitis stuhlmanni*) population in the Kakamega Forest, western Kenya (0°17′30″ N 34°51′22″ E). This rain forest comprises mainly old secondary and near-natural mixed plantation forest [42]. It contains a high density of blue monkeys, approximately 192 individuals/km^2^ [43]. The population has been studied since 1979, with near-daily monitoring beginning in 1997 and focal follows of adult females beginning in 2006 [27]. All individuals in the study groups were individually recognizable based on natural features.

### (b) Data collection

Throughout the study period, a team of trained observers monitored the various groups on a near-daily basis for at least part of the day. Observers kept census records, noting which individuals were present in the group each day. They also recorded all observed agonistic interactions, documenting winners and losers when the interaction had a clear outcome, i.e. one of the opponents showed submissive behaviour [44].

The team conducted 30 min focal follows of adult (parous) females, recording the subject’s activity at 1 min intervals. Observers selected subjects to maintain even sampling across females and across morning, midday and afternoon periods. During follows, observers recorded if females engaged in the following activity categories: feeding, locomotion, socializing (grooming) and resting. Observers recorded the identity of any social partner or animal in proximity to the subject. Partners were considered to be in proximity if they were within 1 m when the subject was resting or engaged in grooming, and within 7 m when the subject was feeding. Observers did not record proximity partners when the focal subject was moving.

### (c) Data analysis

To assess how adult females select a post-fission group, we examined which of their ties to other females in the group and to the original group’s resident male persisted through the fission, considering several tie characteristics, such as tie strength, consistency, difference in dominance rank and relatedness, as well as individual characteristics such as a female’s risk of infanticide and her tie strength with the resident male. We quantified the strength of female social ties (or edges in a network) leading up to a group fission by aggregating the focal data for each female the year before fission. The analysis included all pairs of adult (parous) females in the parent group the year before fission, unless the dyad would include a female who was followed for less than half of the year (this could happen if she had her first infant, becoming an adult, more than halfway through the year). The year before fission was the full year before the onset of subgrouping, which was the period during which group members regularly travelled and foraged in two separate parties for at least part of the day. The subgrouping period ended with the first aggressive intergroup encounter between the two newly separated daughter groups, marking the day of fission [27].

To quantify the strength of affiliative social ties, we calculated a dyadic sociality index (DSI) for each adult female pair in a group using data on grooming and resting in proximity collected during the year before subgrouping started. We used the following formula from Thompson & Cords [31]:


(2.1) $$DSI=\frac{1}{2}\left(\frac{G_{ij}}{G_{med}}\right)+\frac{1}{4}\left(\frac{R_{ij\;}}{R_{med}}+\;\frac{R_{ji\;}}{R_{med}}\right)$$

where, *G*_*ij*_ represents the total time spent grooming between individuals *i* and *j* over their total observation time, divided by the median grooming values of all dyads in the same pre-fission group as individuals *i* and *j* in the same year. *R*_*ij*_ is the amount of time individual *i* spent resting in proximity (1 m) with *j* during individual *i*’s observation time, while *R*_*ji*_ is the amount of time individual *j* spent resting in proximity (1 m) with *i* during individual *j*’s observation time. These proximity terms are separated because they are not symmetrical measures. *R*_*med*_ is the median proximity value of all dyads in the same pre-fission group as individuals *i* and *j* in the same year. We used proximity within 1 m to calculate the strength of social ties because this metric varies substantially across dyads: female blue monkeys may rest within 1 m of their preferred partners 100 times longer than with their least preferred partners [45]. A DSI score >1 means the dyad spent more time grooming and resting in proximity than the median value for all female–female dyads in the same group. In cases where a dyad’s DSI was calculated from a single individual’s focal follow data (see the following paragraph), there was only a single proximity term in the equation and we multiplied that term by one-half instead of one-quarter.

To assay a social tie’s consistency, we compared DSI values for all dyads included in the analysis above to the previous year, i.e. to an annual period starting 2 years before the onset of subgrouping. We relaxed eligibility rules to make this calculation. Specifically, three females were not yet adults (and hence not the subject of focal follows) at this time, so their DSIs 2 years before fission were derived only from the focal follows of their social partners. Four other females were the subject of focal follows for less than half of this annual period, so their DSIs were also calculated with less data than usual. We coded dyadic ties as ‘consistently strong’ if the DSI was >1 for both years, as ‘consistently weak’ if the DSI was <1 for both years, and as ‘inconsistent’ if the DSI was >1 in 1 year and <1 in the other. These categories were entered into the analysis as dummy variables, so the model included one binary (yes/no) variable representing if the tie was consistently strong and another representing if the tie was consistently weak. Inconsistent ties thus served as the reference class.

To calculate dominance rank, we aggregated data on agonistic interactions (from ad libitum and focal follow records) to create winner : loser matrices for each calendar year. We used the I&SI (Inconsistencies and Strength of Inconsistencies) method as implemented in ‘Domicalc’ [46], which minimizes the number and strength of inconsistencies of agonistic interactions in an ordinal linear hierarchy [44]. We used a group’s hierarchy calculated for the calendar year in which the fission took place (including agonistic interactions only up to the date of fission completion). We turned rank into a dyadic, non-directional variable, called rank distance, by counting the number of rank steps between two individuals. To facilitate model convergence, we standardized rank distance with a Z-score.

We calculated relatedness between two individuals based on known maternal kinship records, as paternity was not known. For the 59 unique females in the analysis (some were present in two fissions), we knew maternal relations back at least two generations for 42% of them, one generation for 39% and no previous generations for 19%. We assigned mother–offspring dyads a relatedness of 0.5, sister and grandparent–grandchild dyads a relatedness of 0.25, aunt–niece dyads a relatedness of 0.125, cousin dyads a relatedness of 0.0625 and more distant relatives a relatedness of 0.03125. Unrelated individuals and individuals with unknown maternal relatedness received a relatedness value of zero.

We used birth records to infer a female’s reproductive status at the time of fission. Given an average gestation of 176 days [47], we categorized females as pregnant for 176 days before an offspring’s birth. We considered females as lactating if the infant was less than 1 year old at the time of fission, as lactation decreases substantially after the first year of infant life (M. Cords, 2017, unpublished data). If a female’s infant died before 1 year that female was no longer considered to be lactating (until her next birth). We considered a female to be ‘at risk of infanticide’ if she was lactating or pregnant at the time of fission because she either had or would soon have a dependent, vulnerable infant. We used this binary ‘at risk’ categorization to create two datasets and ran the conditional logit model on each of them (see below).

A resident male was designated as any adult male who was the sole male in the group for at least seven consecutive observation days (which sometimes exceeded one calendar week). A male lost his resident status after he was not present in the group at all for seven consecutive observation days [48]. For all five fissions, the resident male had been resident for at least one gestation length (>176 days) at the time of fission [47]. This does not guarantee his paternity, as blue monkey resident males sire only 61% of the offspring in their groups on average [49]. The resident male was never the sole male leading up to fission—other males visited each group during the year before fission. We included a binary variable in the conditional logit model representing whether the original group’s resident male was present in a given post-fission group (residents invariably ended up in one of the two groups). We also calculated each female’s DSI with the original group’s resident male in the year before fission. Given a female’s DSI with a male is calculated only from her focal follow records (males were not focal subjects), we used the adjusted equation outlined above (weighting grooming and resting in proximity equally) and used the median values of grooming and resting in proximity across all female–resident male dyads per pre-fission group.

To assess which adult females stayed together after fission, we ran a separable temporal exponential random graph model (STERGM; R v.4.4.1 [50]; R package *tergm* v.4.2.1 [51]), which allows one to assess how nodal and edge covariates influence the formation, persistence and dissolution of edges in a network over two or more timesteps. If the individuals in a dyad joined the same post-fission group, we considered their tie or edge to have persisted. Accordingly, we modelled edge persistence from the pre-fission network to the post-fission network. The model included four dyadic edge covariates: two dummy variables representing social tie strength and consistency classes, relatedness and rank distance. To understand why some kin dyads stay together post-fission while others break apart, we added infanticide risk as a nodal trait in the model and included an interaction term between homophily (having the same attribute) of that trait and the edge covariate relatedness, thus testing whether more closely related kin dyads are more likely to persist if they have the same risk of infanticide. We also added an interaction term between relatedness and the tie strength and consistency (dummy) variables to assess whether kin dyads with high DSI scores were more likely to stay together post-fission than ones with low DSI scores. Finally, we included a block-diagonal constraint in the model that limited analysis to dyads of females from the same pre-fission group [52], allowing for the inclusion of all five networks in a single model.

To assess how a female’s risk of infanticide influenced her decision to stay in the group with the original group’s resident male, we ran two conditional logit models (R v.4.4.1 [50]; R package *mclogit* v.0.9.6 [53]). Conditional logit models analyse how actors make decisions by assessing the attributes of each alternative choice [54,55]. We created two datasets, one comprising females at risk of infanticide at the time of fission and one comprising females not at risk. Each female in a given dataset had two entries per fission, one for each post-fission group she could have chosen. These two entries were grouped in the model with a unique ‘choice ID’. The outcome variable for each model represented which choice was selected by a given actor, thus whether (yes/no) the female joined the given post-fission group. We ran a model on each dataset that included a binary predictor expressing whether the original group’s resident male was present in each of the daughter groups. We also included an interaction term between this variable and a covariate that measured the strength of a female’s tie to the original group’s resident male to see if females chose to stay with the resident male if they had associated frequently pre-fission. Given that conditional logit models assess characteristics of alternate choices only, and not characteristics of the decision-maker, tie strength with the resident male could be analysed in the model only as part of an interaction with a covariate that did vary by alternate choice [56].

## 3. Results

Groups fissioned at a mean size of 45.6 ± s.d. 9.6 adult females and juveniles (range: 31–56). The larger post-fission group accounted for a mean of 76% (range: 65–82%) of the pre-fission group’s adult females and juveniles. The age-sex composition of groups was generally similar, proportionally, before versus after fission (electronic supplementary material, table S1). Adult female ages at the time of fission ranged from 6.6 to 27.8 years old (mean: 16.4 ± s.d. 5.7 years).

The STERGM analysis included 770 dyads across the five fissions, of which 101 were kin (13%). Of these kin dyads, 25 split into different post-fission groups across four of the five fissions. More closely related kin dyads, especially mother–offspring, sister and grandparent–granddaughter dyads, had higher median DSI scores a year before fission than unrelated and more distantly related individuals (electronic supplementary material, figure S1). However, of 669 unrelated dyads, 359 (54%) had a DSI >1, and of the 441 dyads that had a DSI >1 the year before fission, 81% comprised unrelated individuals.

**Figure 1.**
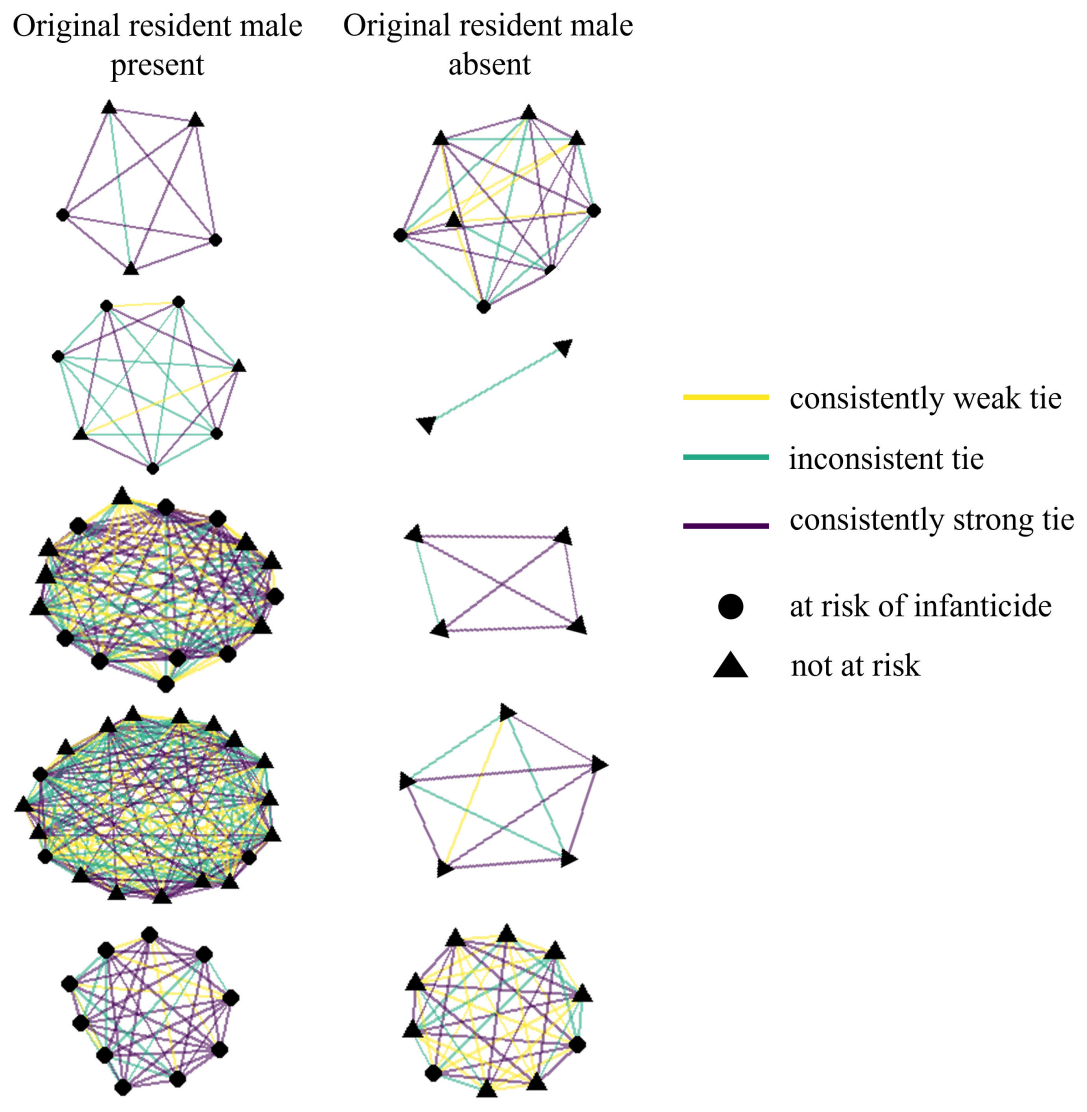
A network representation of the post-fission groups according to their tie strength and consistency in the pre-fission group. Networks placed side by side represent the two post-fission products of a given fission event (*n* = 5), with the left column indicating which post-fission group contained the original group’s resident male and the right column indicating the post-fission group without him. Nodes represent individual adult females, with circles representing those at risk of infanticide and triangles representing those not at risk. Lines represent edges (ties) between females in the network, coloured according to the strength-consistency class of the dyad’s social tie.

The model including interactions between relatedness and the two tie strength-consistency variables (Table S2) had a higher Akaike information criterion (AIC) (1003) than the model without (998), so we proceeded with the model that did not include these interactions as it was a better fit for the data given the ΔAIC [57] (table 1). This model performed better than a null model with no edge or node covariates (AIC: 998 versus 1043). Overall, edges had a 45% probability of persisting from before fission to after fission. Pre-fission edges between females with consistently strong ties had greater odds of persisting after fission than those between females with inconsistent ties (table 1), but there was no evidence that consistently strong ties were more likely to persist than consistently weak ties (electronic supplementary material, table S3). Consistently weak ties, in turn, showed a similar trend of being more likely to persist after fission than inconsistent ties (table 1, figure 1). Edges between females of similar infanticide risk also had greater odds of persisting after fission than those with different risk. Edges between more closely related females had greater odds of persisting after fission only when the two females had the same infanticide risk. There was no evidence indicating that the rank distance between two females influenced the odds of their edge persisting after fission.

**Table 1.** STERGM model results assessing how edge and nodal covariates affected the odds of an edge (female–female tie) persisting between two timesteps, before versus after fission. Reference category is indicated in parentheses. *P*-values < 0.05 are bolded.

predictors	odds ratio	s.e.	*p*
baseline edge persistence	0.81	0.21	0.31
consistently strong tie (inconsistent tie)	1.80	0.18	**0.001**
consistently weak tie (inconsistent tie)	1.41	0.20	0.08
relatedness	1.23	1.12	0.85
infanticide risk (dissimilar risk)	1.78	0.16	**<0.001**
relatedness × infanticide risk	147.01	2.14	**0.02**
rank distance—standardized	0.98	0.01	0.28

**Figure 2.**
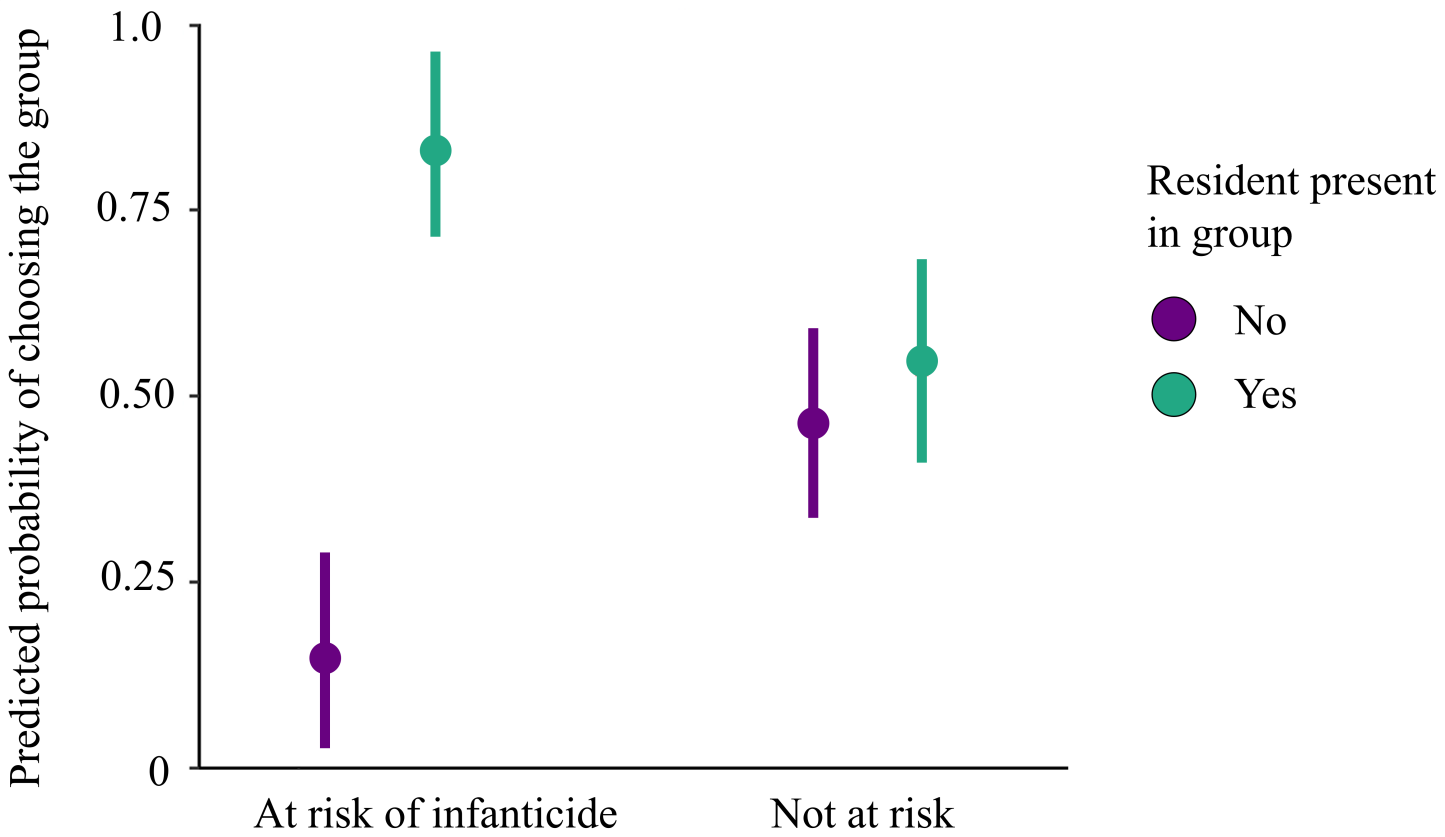
Predicted probability of whether a female would join the post-fission group with the original group’s resident male depending on whether she was at risk of infanticide when fission occurred. Each female (at risk: *n* = 35; not at risk: *n* = 51) has two data points in the model, one per potential group she could join. Bars represent 95% confidence intervals.

The conditional logit models included 86 adult females from five fission events. Of these 86 females, 57 (66%) ended up in the group with the original group’s resident male. At the time of fission, 35 of 86 females (41%) were at risk of infanticide (either pregnant or with a dependent infant), 29 of which (83%) ended up in the group with the original male resident. Females who had or would soon have a dependent infant at the time of fission were significantly more likely to join the post-fission group that contained the original group’s resident male than the group without him (odds ratio (OR) = 3.63, *z* = 2.09, *p* = 0.04). For females not facing infanticide risk, however, we found no evidence that the original male resident’s presence in a post-fission group influenced their choices (OR = 1.36, *z* = 0.86, *p* = 0.39; figure 2). There was also no evidence that tie strength (DSI) with the resident influenced post-fission group choice of females at risk of infanticide (OR = 1.25, *z* = 0.60, *p* = 0.55) or those not at risk (OR = 0.86 , *z* = −0.51, *p* = 0.61).

All post-fission groups that included the original group’s resident male did not experience their next takeover for at least a year. Post-fission groups that did not include the original resident male invariably had at least one new male join on their first day as a new group, and a new resident male was established shortly thereafter (mean of 30.8 ± s.d. 26.4 days post-fission). Of the six infants that moved into the post-fission group without the resident male, five survived to 1 year old (83%). The infant that died was the first infant born (18 days) after the takeover. Infant survival was higher in the post-fission group with the resident male, where 33 of 35 infants who joined that group (94%) survived to 1 year old. One of the two infants that were lost disappeared with its mother and probably did not die through infanticide.

## 4. Discussion

Female blue monkeys chose their post-fission group based on multiple aspects of their social ties. First, females with consistently strong affiliative ties across the 2 years before fission were more likely to stay together than females with inconsistent tie strength. There was also some evidence that females with consistently weak ties were more likely to stay together than inconsistent ones. However, it was not clear that females with consistently strong ties were more likely to stay together than those with consistently weak ones. This result aligns with the conclusions of Thompson & Cords [31], who found that females with strong, consistent ties from year to year had the greatest longevity and that females whose strongest ties were inconsistent across years had the highest risk of death, even higher than females with consistently weak ties. Coupled with the insights from Thompson & Cords (2018), evidence that females with consistently strong ties stayed together during group fission supports the hypothesis that females specifically prioritize relationships that can provide advantages to their fitness, and highlights that not all affiliative ties are equally advantageous. Furthermore, even though stronger ties characterize more closely related individuals in blue monkeys (electronic supplementary material, figure S1; [27,28]), being more closely related did not predict whether consistently strong or consistently weak tied females remained together after fission, evident in the fact that the interaction between tie strength-consistency and relatedness weakened the initial model. These results indicate that females chose the post-fission group in which they could maintain strong affiliative ties with both related and unrelated individuals.

Females that were pregnant or with a dependent infant at the time of fission were more likely to join the post-fission group that contained the original group’s resident male than the group without him. The presence of the original group’s resident male did not appear to influence which group a female with no risk selected. These different results suggest that post-fission group choice may be part of an adaptive anti-infanticide strategy. The resident male of each group at the time of fission had been resident long enough to have sired these females’ offspring and takeover by a novel male occurred soon after fission in each daughter group without the original resident. Infanticide is associated with the arrival of novel males [36], so females are likely to minimize their risk of infant loss by staying with the original group’s resident male. The infant that died in the group without the resident male was the first to be born after takeover by a novel male. The fact that this infant died, while later born infants did not, is consistent with the idea that the new male could identify this infant as not his own offspring, while those born later had more ambiguous paternity and were less at risk. Studies in other cercopithecine primates have also illustrated that maintaining affiliative ties with males can provide benefits for lactating females, including protection against infanticide and harassment [37, 38].

There was no evidence that two females were more likely to stay together after fission based on degree of relatedness unless they shared the same infanticide risk, emphasizing that females’ individual attributes can influence which aspects of their social ties they prioritized. This finding is consistent with other studies showing that females that split from matrilineal kin typically do prioritize other factors in their decision-making. For example, savannah baboons with few maternal kin preferred to stay close to paternal kin and non-kin affiliates [4]. In macaques, matrilines were more likely to be broken if the females in the matriline were very low ranking [58]. Furthermore, this result reveals that relatedness alone does not guarantee that females will stay together after group fission and that strong affiliation is a more important consideration in post-fission decision-making. Studies of other female-bonded animals have reported that kin generally stay together after group fission [4–6,13]. Kin-based affiliation is suggested to be adaptive due to increased cooperation, tolerance and long-lived social bonds [1,28,39]. These mechanisms suggest that affiliative social ties are an important prerequisite to gain benefits from kin and that relatedness alone is not sufficient to make ties beneficial.

If rank influenced post-fission group choice, such that low-ranking individuals could improve their status in a new group, we expected to see that individuals of more disparate ranks would be least likely to stay together after fission. We found no evidence, however, that rank distance between individuals influenced the likelihood that they stayed together after fission. Improving rank appears not to be a motivation for choosing a particular post-fission group in blue monkeys. This inference is also consistent with prior studies of this population showing that female dominance rank does not predict survival, fertility or nutrient balancing [31,33,34]. Given the lack of evidence for advantages of high dominance rank in blue monkeys, this result also supports the hypothesis that only relationships affecting fitness influenced post-fission group choice.

## 5. Conclusion

By assessing which social ties are maintained during fission, this study sheds light on which relationships are most beneficial to individuals living in groups. Both a female’s individual traits (reproductive status) and her social ties (intra and intersexual) were influential in shaping post-fission group choice. Ultimately, adult females chose to maintain social relationships that are likely to benefit their survival and reproduction, and they appeared to account for multiple types of relationships simultaneously. Social decision-making in blue monkeys is evidently a complex, adaptive process influenced by both social ties and attributes of the decision-maker. Our findings underscore the importance of evaluating concurrently the multiple relevant criteria that could influence an animal’s decision-making process in a social context.

## Data Availability

The data and code used in this study can be found on Dryad [59]. Supplementary material is available online [60].
